# The Electromechanical Behavior of a Micro-Ring Driven by Traveling Electrostatic Force

**DOI:** 10.3390/s120201170

**Published:** 2012-01-30

**Authors:** Xiuqian Ye, Yibao Chen, Da-Chih Chen, Kuo-Yi Huang, Yuh-Chung Hu

**Affiliations:** 1 School of Electromechanical Automobile Engineering, Yantai University, 32 Qingquan Road, Yantai 264005, China; E-Mails: xiuqian868@yahoo.com (X.Y.); yibaoch@yahoo.com.cn (Y.C.); 2 Department of Mechanical and Electromechanical Engineering, National ILan University, 1 Section, Shen-Lung Road, ILan 260, Taiwan; E-Mail: dcchen@niu.edu.tw; 3 Department of Bio-Industrial Mechatronics Engineering, National Chung Hsing University, 150 Kuo Kuang Road, Taichung 402, Taiwan; E-Mail: kuoyi@dragon.nchu.edu.tw

**Keywords:** electrostatics, MEMS, microstructures, stabilities, traveling forces

## Abstract

There is no literature mentioning the electromechanical behavior of micro structures driven by traveling electrostatic forces. This article is thus the first to present the dynamics and stabilities of a micro-ring subjected to a traveling electrostatic force. The traveling electrostatic force may be induced by sequentially actuated electrodes which are arranged around the flexible micro-ring. The analysis is based on a linearized distributed model considering the electromechanical coupling effects between electrostatic force and structure. The micro-ring will resonate when the traveling speeds of the electrostatic force approach some critical speeds. The critical speeds are equal to the ratio of the natural frequencies to the wave number of the correlative natural mode of the ring. Apart from resonance, the ring may be unstable at some unstable traveling speeds. The unstable regions appear not only near the critical speeds, but also near some fractions of some critical speeds differences. Furthermore the unstable regions expand with increasing driving voltage. This article may lead to a new research branch on electrostatic-driven micro devices.

## 1. Introduction

The principles of electrostatic forces are very commonly used in micro actuating/sensing devices because of the advantages rapid response, low power consumption, compatibility with the fabrication process of integrated circuits (ICs), and being integrable with ICs. The electrostatic driving principle consists of the coupling of two energy domains, namely the electrical and mechanical energy domains. It is very challenging to accurately model electrostatic microstructures because of their nonlinear electromechanical coupling behavior. Furthermore effects such as non-ideal boundary conditions, fringing fields, the pre-deformation induced by initial stresses, and non-homogeneous structures further complicate the modeling. A review paper [10] presented an overview of the existing techniques applied to the MEMS electrostatic actuation modeling before 2005 and the dynamic behavior of the electromechanical system. Another review paper [2] provided an overview of the fundamental research before 2008 on nonlinear behaviors of electrostatic-driven microresonators, including direct and parametric resonances, parametric amplification, impacts, self-excited oscillations, and collective behaviors, such as localization and synchronization, which arise in coupled resonator arrays. Other review papers [3,4] present an overview of the existing analytical models before 2007 for electrostatically actuated microdevices. The author Hu had published a review paper [5] which introduced the techniques before 2010 for the physical model of pull-in voltage, dynamic characteristic analysis, air damping effect, reliability, numerical modeling method, and application of electrostatic-driven MEMS devices. Furthermore the effects of non-ideal boundary conditions, fringing fields, the pre-deformation induced by initial stresses, and non-homogeneous structures were also detailed in that review paper. The dynamic instability of a microstructure driven by alternative electrostatic forces was published by the author Hu [6] for the first time in 2004. After that more and more literature about the dynamic instability of a microstructure driven by electrostatic forces was published. After surveying the large number of correlative literatures mentioned in the aforesaid literatures, we find that there is no literature investigating the electromechanical behavior of a microstructure driven by traveling electrostatic forces. Therefore this paper presents for the first time the study of the dynamics and stabilities of microstructures driven by traveling electrostatic forces.

We develop a theoretical model of a micro-ring driven by a radial electrostatic force which travels around the circumference of the micro-ring. The micro-ring model is based on the theory of vibrations of a circular ring [7]. The traveling electrostatic force is modeled as a concentrated electrostatic force in the radial direction which travels around the circumference of the micro-ring. The magnitude of the traveling electrostatic force is proportional to the square of the driving voltage and inversely proportional to the square of the gap between the micro-ring and the driving electrodes [6]. The moving feature of the traveling concentrated electrostatic force is modeled based on the author Hu’s published work [8], namely as a Dirac delta function whose location varies with the traveling speed. First of all, a linearized dimensionless governing equation is derived based on the aforesaid assumptions, which is a linear partial differential equation with periodically time-varying coefficient. For the geometrical periodicity of the circular ring, we make an inspired guess of the deflection function to discretize the governing equation, which results in a set of linear ordinary differential equations with periodically time-varying coefficients. Then we solve the discretized governing equation by the Runge-Kutta numerical integration method, which is available in the commercial software MATLAB. The dynamics and stabilities of the micro-ring are both studied in this article. The Floquet theory [9,10] is used to determine whether the micro-ring is stable or not.

## 2. Theoretical Modeling

### 2.1. Linearized Non-Dimensional Governing Equation

**Figure 1.**
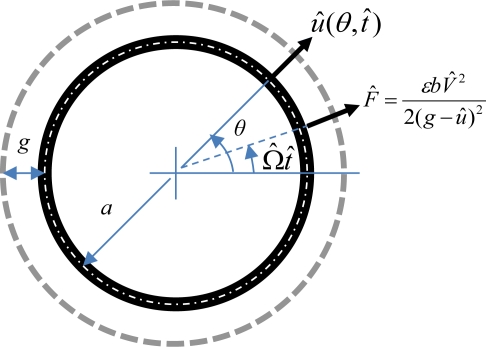
The schematic diagram of a flexible micro-ring driven by a traveling electrostatic force.

Figure 1 shows a flexible micro-ring of radius *a* surrounded by a circle of fixed-electrodes between them a gap of *g*. A bias voltage *V̂* applied sequentially on the fixed-electrodes sets up an electrostatic force *F̂* (*= εbV̂*^2^/2(*g* − *û*)^2^) per unit length traveling at an angular speed of Ω̂ around the circumference of the micro-ring in the radial direction. The micro-ring, when subjected to the traveling electrostatic force, will oscillate with respect to its neutral axis at the deflection of *û*(*θ*,*t̂*). Based on the assumptions that the micro-ring is thin with respect to its radius and that deflection is reasonably small, and if there is no circumferential forcing and if its circumferential inertia term can be negligible [6–8], then its equation of motion in a polar coordinate system is:

(1) $$\rho A\frac{\partial^{2}\hat{u}}{\partial {\hat{t}}^{2}}+\frac{\mathrm{EI}}{a^{4}}\left(\frac{\partial^{4}\hat{u}}{\partial \theta^{4}}+2\frac{\partial^{2}\hat{u}}{\partial \theta^{2}}+\hat{u}\right)=\delta \left(\theta -\hat{\Omega }\hat{t}\right)\frac{\varepsilon b{\hat{V}}^{2}}{2{\left(g-\hat{u}\right)}^{2}}$$

where *A*, *b*, *E*, *I*, *ε*, and *ρ* are the cross-sectional area, width, Young’s modulus, inertia moment of the cross-sectional area, permittivity of free-space, and density of the micro-ring, respectively, while *t̂* is the time. If the fixed electrodes and their gaps are very small compared to the length of the micro-ring, one can simulate the sequentially-actuated electrostatic force as a continuously traveling electrostatic force like the term on the right-hand side of Equation (1) where *δ*(*θ* − Ω̂*t̂*) is the Dirac delta function.

Here we introduce the following dimensionless variables:

(2) $$u=\hat{u}/g,\;t=\hat{t}/T,\;\;\Omega =\hat{\Omega }T,\;V^{2}=a^{4}\varepsilon b{\hat{V}}^{2}/{\mathrm{EIg}}^{2}$$

where *T* is a time-scale defined as $T=\sqrt{\rho Aa^{4}/\mathrm{EI}}$. Rewrite the equation of motion in dimensionless variables:

(3) $$\frac{\partial^{2}u}{\partial t^{2}}+\frac{\partial^{4}u}{\partial \theta^{4}}+2\frac{\partial^{2}u}{\partial \theta^{2}}+u=\delta \left(\theta -\Omega t\right)\frac{V^{2}}{{\left(1-u\right)}^{2}}$$

Expanding the nonlinear term of the electrostatic force by Taylor’s series about the initial equilibrium position of the micro-ring, say *u* = 0:

(4) $$\frac{1}{{\left(1-u\right)}^{2}}=1+2u+3u^{2}+4u^{3}+\cdots$$

and neglecting the second- and higher-order terms based on the assumption of small deflection because the second- and higher-order terms are much smaller than the first-order term, this results in a linear periodically time-varying system:

(5) $$\left(\frac{\partial^{2}u}{\partial t^{2}}+\frac{\partial^{4}u}{\partial \theta^{4}}+2\frac{\partial^{2}u}{\partial \theta^{2}}+u\right)-2\delta \left(\theta -\Omega t\right)V^{2}\;u=\delta \left(\theta -\Omega t\right)V^{2}$$

Let us inspect Equation (5); the terms in the first bracket are attributed to the mechanical characteristics of the micro-ring, while the second term, −2*δ*(*θ* − Ω*t*)*V*^2^, is attributed to the electrostatic force, which shows that the dynamical characteristics of the micro-ring will be altered by the driving voltage *V* as well as the traveling speed Ω.

### 2.2. Discretized Governing Equation

For the geometrical periodicity of a circular ring, one can make an inspired guess of the deflection function:

(6) $$u(\theta ,t)=\sum_{k=1}^{n}\left[\alpha_{k}(t)\text{cos}\;k\theta +\beta_{k}(t)\text{sin}\;k\theta \right]$$

where *α_k_*(*t*) and *β_k_*(*t*), the modal participation factors, are unknowns and have to be determined while the trigonometric functions refer to the natural modes of the ring. In a mathematical sense, the natural modes represent orthogonal vectors that satisfy the boundary conditions of the ring. In cases of finite-degree-of-freedom systems, the vector space is of finite dimension and the number of vectors or natural modes is equal to the number of degrees of freedom. For continuous systems, such as ring, the number of degrees of freedom is infinite. This means that the general solution will be an infinite series. Substituting Equation (6) in Equation (5) gives:

(7) $$\begin{array}{l} \sum_{k=1}^{n}\;\left[{\ddot{\alpha }}_{k}(t)+{\left(k^{2}-1\right)}^{2}\alpha_{k}(t)-2\delta \left(\theta -\Omega t\right)V^{2}\alpha_{k}(t)\right]\text{cos}\;k\theta \\ +\sum_{k=1}^{n}\;\left[{\ddot{\beta }}_{k}(t)+{\left(k^{2}-1\right)}^{2}\beta_{k}(t)-2\delta \left(\theta -\Omega t\right)V^{2}\beta_{k}(t)\right]\text{sin}\;k\theta =\delta \left(\theta -\Omega t\right)V^{2} \end{array}$$

Since the sine and cosine functions are orthogonal, we may proceed as in a Fourier analysis. We multiply the equation on both sides by cos *mθ* and sin *mθ*, where *m*, in general, is either equal to *k* or not:

(8) $$\begin{array}{l} \sum_{k=1}^{n}\;\left[{\ddot{\alpha }}_{k}(t)+{\left(k^{2}-1\right)}^{2}\alpha_{k}(t)-2\delta \left(\theta -\Omega t\right)V^{2}\alpha_{k}(t)\right]\text{cos}\;k\theta \;\text{cos}\;m\theta \\ +\sum_{k=1}^{n}\;\left[{\ddot{\beta }}_{k}(t)+{\left(k^{2}-1\right)}^{2}\beta_{k}(t)-2\delta \left(\theta -\Omega t\right)V^{2}\beta_{k}(t)\right]\text{sin}\;k\theta \;\text{cos}\;m\theta \\ =\delta \left(\theta -\Omega t\right)V^{2}\;\text{cos}\;m\theta \end{array}$$

(9) $$\begin{array}{l} \sum_{k=1}^{n}\;\left[{\ddot{\alpha }}_{k}(t)+{\left(k^{2}-1\right)}^{2}\alpha_{k}(t)-2\delta \left(\theta -\Omega t\right)V^{2}\alpha_{k}(t)\right]\;\text{cos}\;k\theta \;\text{sin}\;m\theta \\ +\sum_{k=1}^{n}\;\left[{\ddot{\beta }}_{k}(t)+{\left(k^{2}-1\right)}^{2}\beta_{k}(t)-2\delta \left(\theta -\Omega t\right)V^{2}\beta_{k}(t)\right]\;\text{sin}\;k\theta \;\text{sin}\;m\theta \\ =\delta \left(\theta -\Omega t\right)V^{2}\;\text{sin}\;m\theta \end{array}$$

Integrating Equations (8) and (9) over the ring circumference gives:

(10) $$\begin{array}{l} \sum_{k=1}^{n}\;\left[{\ddot{\alpha }}_{k}(t)+{\left(k^{2}-1\right)}^{2}\alpha_{k}(t)\right]\int_{0}^{2\pi }\text{cos}\;k\theta \;\text{cos}\;m\theta d\theta -\sum_{k=1}^{n}2V^{2}\alpha_{k}(t)\int_{0}^{2\pi }\delta \left(\theta -\Omega t\right)\text{cos}\;k\theta \;\text{cos}\;m\theta d\theta \\ +\sum_{k=1}^{n}\;\left[{\ddot{\beta }}_{k}(t)+{\left(k^{2}-1\right)}^{2}\beta_{k}(t)\right]\int_{0}^{2\pi }\text{sin}\;k\theta \;\text{cos}\;m\theta d\theta -\sum_{k=1}^{n}2V^{2}\beta_{k}(t)\int_{0}^{2\pi }\delta \left(\theta -\Omega t\right)\text{sin}\;k\theta \;\text{cos}\;m\theta d\theta \\ =\int_{0}^{2\pi }\delta \left(\theta -\Omega t\right)V^{2}\;\text{cos}\;m\theta d\theta \end{array}$$

(11) $$\begin{array}{l} \sum_{k=1}^{n}\;\left[{\ddot{\alpha }}_{k}(t)+{\left(k^{2}-1\right)}^{2}\alpha_{k}(t)\right]\int_{0}^{2\pi }\text{cos}\;k\theta \;\text{sin}\;m\theta d\theta -\sum_{k=1}^{n}2V^{2}\alpha_{k}(t)\int_{0}^{2\pi }\delta \left(\theta -\Omega t\right)\text{cos}\;k\theta \;\text{sin}\;m\theta d\theta \\ +\sum_{k=1}^{n}\;\left[{\ddot{\beta }}_{k}(t)+{\left(k^{2}-1\right)}^{2}\beta_{k}(t)\right]\int_{0}^{2\pi }\text{sin}\;k\theta \;\text{sin}\;m\theta d\theta -\sum_{k=1}^{n}2V^{2}\beta_{k}(t)\int_{0}^{2\pi }\delta \left(\theta -\Omega t\right)\text{sin}\;k\theta \;\text{sin}\;m\theta d\theta .\\ =\int_{0}^{2\pi }\delta \left(\theta -\Omega t\right)V^{2}\;\text{sin}\;m\theta d\theta \end{array}$$

Using the following orthogonality conditions of trigonometric functions and the characteristics of the Dirac delta function:

(12) $$\int_{0}^{2\pi }\text{cos}\;k\theta \;\text{cos}\;m\theta \;d\theta =\int_{0}^{2\pi }\text{sin}\;k\theta \;\text{sin}\;m\theta \;d\theta =\{\begin{array}{c} \pi ,\;\;\;\;\text{for}\;k=m\\ 0,\;\;\;\;\text{for}\;k\neq m \end{array}$$

(13) $$\int_{0}^{2\pi }\text{sin}\;k\theta \;\text{cos}\;m\theta \;d\theta =\int_{0}^{2\pi }\text{cos}\;k\theta \;\text{sin}\;m\theta \;d\theta =0$$

(14) $$\{\begin{array}{c} \int_{0}^{2\pi }\delta (\theta -\Omega t)\;\text{cos}\;k\theta \;\text{cos}\;m\theta \;d\theta =\text{cos}\;k\Omega t\;\text{cos}\;m\Omega t\\ \int_{0}^{2\pi }\delta (\theta -\Omega t)\;\text{sin}\;k\theta \;\text{cos}\;m\theta \;d\theta =\text{sin}\;k\Omega t\;\text{cos}\;m\Omega t\\ \int_{0}^{2\pi }\delta (\theta -\Omega t)\;\text{cos}\;k\theta \;\text{sin}\;m\theta \;d\theta =\text{cos}\;k\Omega t\;\text{sin}\;m\Omega t\\ \int_{0}^{2\pi }\delta (\theta -\Omega t)\;\text{sin}\;k\theta \;\text{sin}\;m\theta \;d\theta =\text{sin}\;k\Omega t\;\text{sin}\;m\Omega t \end{array}$$

(15) $$\{\begin{array}{c} \int_{0}^{2\pi }\delta (\theta -\Omega t)V^{2}\text{cos}\;m\theta \;d\theta =V^{2}\text{cos}\;m\Omega t\\ \int_{0}^{2\pi }\delta (\theta -\Omega t)V^{2}\text{sin}\;m\theta \;d\theta =V^{2}\text{sin}\;m\Omega t \end{array}$$

we are able to simplify Equations (10) and (11) in the matrix form:

(16) $${\left[I\right]}_{2n\times 2n}{\left\{\ddot{X}\right\}}_{2n\times 1}+\left(\left[\begin{array}{cc} {\left[K^{s}\right]}_{n\times n} & {\left[0\right]}_{n\times n}\\ {\left[0\right]}_{n\times n} & {\left[K^{s}\right]}_{n\times n} \end{array}\right]-\left[\begin{array}{cc} {\left[K^{e1}\right]}_{n\times n} & {\left[K^{e2}\right]}_{n\times n}\\ {\left[K^{e2}\right]}_{n\times n}^{T} & {\left[K^{e3}\right]}_{n\times n} \end{array}\right]\right){\left\{X\right\}}_{2n\times 1}={\left\{Q\right\}}_{2n\times 1}$$

Since Equation (16) is of the form of a *n* degree of freedom oscillator equation, it has become customary to view this equation in terms of modal mass, stiffness, and forcing, where [**I**] is an identity modal mass matrix of order 2*n*, [**K***^s^*] is the structural modal stiffness matrix of order *n*, [**K***^e^*^1^], [**K***^e^*^2^], and [**K***^e^*^3^] are the *n*-th order electrical modal stiffness matrices attributed to the traveling electrostatic forces, {**X**} is the general coordinates, {**Q**} is the generalized forcing function. Their elements are given as the following:

(17) $${\left\{X\right\}}^{T}=\left\{\begin{array}{cccccccc} \alpha_{1}(t) & \alpha_{2}(t) & \cdots & \alpha_{n}(t) & \beta_{1}(t) & \beta_{2}(t) & \cdots & \beta_{n}(t) \end{array}\right\}$$

(18) $${\left\{Q\right\}}^{T}=V^{2}\left\{\begin{array}{cccccccc} \text{cos}\;\Omega t & \text{cos}\;2\Omega t & \cdots & \text{cos}\;n\Omega t & \text{sin}\;\Omega t & \text{sin}\;2\Omega t & \cdots & \text{sin}\;n\Omega t \end{array}\right\}$$

(19) $$K_{\mathrm{ij}}^{s}=\{\begin{array}{cc} i^{2}-1, & \text{for}\;i=j.\\ 0, & \text{for}\;i\neq j. \end{array}$$

(20) $$K_{\mathrm{ij}}^{e1}=V^{2}\text{cos}\;i\Omega t\;\text{cos}\;j\Omega t=V^{2}\frac{\text{cos}(i-j)\Omega t+\text{cos}(i+j)\Omega t}{2}$$

(21) $$K_{\mathrm{ij}}^{e2}=V^{2}\;\text{cos}\;i\Omega t\;\text{sin}\;j\Omega t=V^{2}\;\frac{\text{sin}(i+j)\Omega t-\text{sin}(i-j)\Omega t}{2}$$

(22) $$K_{\mathrm{ij}}^{e3}=V^{2}\;\text{sin}\;i\Omega t\;\text{sin}\;j\Omega t=V^{2}\;\frac{\text{cos}(i-j)\Omega t-\text{cos}(i+j)\Omega t}{2}$$

Equation (16) reveals that the total stiffness matrix is the difference of the structural stiffness matrix and the electrical stiffnes matrix, *i.e.*,

(23) $${\left[K\right]}_{2n\times 2n}=\left[\begin{array}{cc} \left[K^{1}\right] & \left[K^{2}\right]\\ {\left[K^{2}\right]}^{T} & \left[K^{3}\right] \end{array}\right]=\left[\begin{array}{cc} \left[K^{s}\right] & \left[0\right]\\ \left[0\right] & \left[K^{s}\right] \end{array}\right]-\left[\begin{array}{cc} \left[K^{e1}\right] & \left[K^{e2}\right]\\ {\left[K^{e2}\right]}^{T} & \left[K^{e3}\right] \end{array}\right]=\left[\begin{array}{cc} \left[K^{s}]-[K^{e1}\right] & -[K^{e2}]\\ -{\left[K^{e2}\right]}^{T} & \left[K^{s}]-[K^{e3}\right] \end{array}\right]$$

From the Equations (19) to (23), we know that [**K**^1^] and [**K**^3^] are symmetrical while [**K**^2^] is not. The total stiffness matrix is a periodically time-varying function whose oscillation is proportional to *V*^2^ and frequencies are (*i* – *j*) and (*i* + *j*) times the traveling speed Ω.

## 3. Free Vibration of the Ring Structure

Consider the free vibration of the ring, namely the homogeneous part of Equation (3):

(24) $$\frac{\partial^{2}u}{\partial t^{2}}+\frac{\partial^{4}u}{\partial \theta^{4}}+2\frac{\partial^{2}u}{\partial \theta^{2}}+u=0$$

For the case of free vibration, Equation (15) is simplified to:

(25) $$\left[\begin{array}{cc} 1 & 0\\ 0 & 1 \end{array}\right]\left\{\begin{array}{c} {\ddot{\alpha }}_{k}(t)\\ {\ddot{\beta }}_{k}(t) \end{array}\right\}+\left[\begin{array}{cc} {\left(k^{2}-1\right)}^{2} & 0\\ 0 & {\left(k^{2}-1\right)}^{2} \end{array}\right]\left\{\begin{array}{c} \alpha_{k}(t)\\ \beta_{k}(t) \end{array}\right\}=\left\{\begin{array}{c} 0\\ 0 \end{array}\right\}$$

where *k* = 1 ∼ *n*. For no external force, substituting:

(26) $$\left\{\begin{array}{c} \alpha_{k}(t)\\ \beta_{k}(t) \end{array}\right\}=\left\{\begin{array}{c} {\bar{\alpha }}_{k}\\ {\bar{\beta }}_{k} \end{array}\right\}\;e^{i\omega_{k}t}$$

gives:

(27) $$\left[\begin{array}{cc} {\left(k^{2}-1\right)}^{2}-\omega_{k}^{2} & 0\\ 0 & {\left(k^{2}-1\right)}^{2}-\omega_{k}^{2} \end{array}\right]\left\{\begin{array}{c} {\bar{\alpha }}_{k}\\ {\bar{\beta }}_{k} \end{array}\right\}=\left\{\begin{array}{c} 0\\ 0 \end{array}\right\}$$

Since, in general, *ᾱ_k_* and *β̄_k_* are both nonzero, it must be that the determinant is zero, namely the eigenvalues and the correlative eigenvectors are the natural modes of the ring. Thus, for each vale of *k* which also refers to the wave number, we encounter a dimensionless natural frequency:

(28) $$\omega_{k}^{2}={\left(k^{2}-1\right)}^{2}$$

and the correlative mode shape function, namely the natural mode, *U_k_*(θ):

(29) $$U_{k}(\theta )=\text{cos}\;k\theta +\text{sin}\;k\theta$$

**Figure 2.**
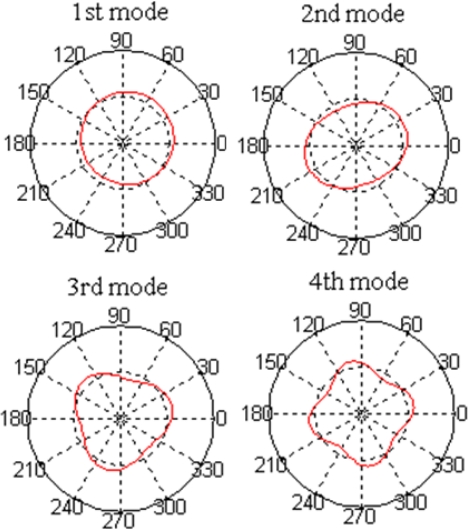
The first four natural modes (mode shape functions) of ring: The first mode is a rigid-body mode while the others are flexible modes.

Figure 2 shows the first four natural modes (*k* = 1 ∼ 4) which are obtained from Equation (29). The first natural mode is a rigid-body mode whose natural frequency is zero and the others are flexible modes.

## 4. Dynamic Response

### 4.1. The Dynamic Response of a Traveling Constant Force

First of all, let us consider the case of a ring driven by a constant force traveling around its circumference. Then Equation (3) is simplified to:

(30) $$\frac{\partial^{2}u}{\partial t^{2}}+\frac{\partial^{4}u}{\partial \theta^{4}}+2\frac{\partial^{2}u}{\partial \theta^{2}}+u=\delta \left(\theta -\Omega t\right)F$$

where *F* is a dimensionless force, and Equation (16) is simplified to:

(31) $$\left[\begin{array}{cc} 1 & 0\\ 0 & 1 \end{array}\right]\left\{\begin{array}{c} {\ddot{\alpha }}_{k}\\ {\ddot{\beta }}_{k} \end{array}\right\}+\left[\begin{array}{cc} {\left(k^{2}-1\right)}^{2} & 0\\ 0 & {\left(k^{2}-1\right)}^{2} \end{array}\right]\left\{\begin{array}{c} \alpha_{k}\\ \beta_{k} \end{array}\right\}=\left\{\begin{array}{c} \frac{F}{\pi }\text{cos}\;k\Omega t\\ \frac{F}{\pi }\text{sin}\;k\Omega t \end{array}\right\}$$

where *k* = 1 ∼ *n*. The solution of Equation (31) is:

(32) $$\alpha_{k}(t)=\frac{F}{\pi }\frac{\text{cos}\;k\Omega t}{{\left(k^{2}-1\right)}^{2}-{\left(k\Omega \right)}^{2}},\;\;\beta_{k}(t)=\frac{F}{\pi }\frac{\text{sin}\;k\Omega t}{{\left(k^{2}-1\right)}^{2}-{\left(k\Omega \right)}^{2}}$$

Substituting Equation (32) into Equation (6), we get the response of the ring:

(33) $$u(\theta ,t)=\frac{F}{\pi }\sum_{k=1}^{n}\frac{\text{cos}\;k(\Omega t-\theta )}{{\left(k^{2}-1\right)}^{2}-{\left(k\Omega \right)}^{2}}$$

The response at the position of the traveling constant force is:

(34) $$u(\Omega t,t)=\sum_{k=1}^{n}\frac{F/\pi }{{\left(k^{2}-1\right)}^{2}-{\left(k\Omega \right)}^{2}}$$

When the traveling speeds approach the values which make the denominators in Equation (34) equal to zero, the ring is resonant and that speed is called the critical speed Ω_cr_. By equating the denominators in Equation (34) to zero, we can obtain the critical speeds corresponding to each mode:

(35) $${\left(k^{2}-1\right)}^{2}-{\left(k\Omega_{\mathrm{cr}}\right)}^{2}=0\;\Rightarrow \;\Omega_{\mathrm{cr},k}=\frac{k^{2}-1}{k}=\frac{\omega_{k}}{k}$$

where ω*_k_* = *k*^2^ − 1 is the dimensionless natural frequency of the *k*^th^ mode of the ring. Equation (35) reveals that the ring will resonate when the traveling speeds approach to the one-*k*^th^ of the natural frequency of the *k*^th^ mode.

### 4.2. The Dynamic Response of a Traveling Electrostatic Force

To obtain the dynamic response of Equation (16), we have to use numerical integration because it is a periodically time-varying system. To solve by numerical integration, one has to transform Equation (16) into state space. We define the state vector as:

(36) $${\left\{Y\right\}}_{4n\times 1}^{T}=\left\{\begin{array}{cc} {\left\{X\right\}}^{T} & {\left\{\dot{X}\right\}}^{T} \end{array}\right\}$$

and then transform Equation (16) the into state space as:

(37) $${\left\{\dot{Y}\right\}}_{4n\times 1}={\left[A\right]}_{4n\times 4n}{\left\{Y\right\}}_{4n\times 1}+{\left[B\right]}_{4n\times 4n}{\left\{F\right\}}_{4n\times 1}$$

where:

(38) $$\left[A\right]=\left[\begin{array}{cc} {\left[0\right]}_{2n\times 2n} & {\left[I\right]}_{2n\times 2n}\\ -{\left[K\right]}_{2n\times 2n} & {\left[0\right]}_{2n\times 2n} \end{array}\right]$$

(39) $$\left[B\right]=\left[\begin{array}{cc} {\left[0\right]}_{2n\times 2n} & {\left[0\right]}_{2n\times 2n}\\ {\left[0\right]}_{2n\times 2n} & {\left[I\right]}_{2n\times 2n} \end{array}\right]$$

(40) $${\left\{F\right\}}_{4n\times 1}=\left\{\begin{array}{c} {\left\{0\right\}}_{2n\times 1}\\ {\left\{Q\right\}}_{2n\times 1} \end{array}\right\}$$

Consequently we can obtain the solution by numerical integration:

(41) $$\left\{Y(t)\right\}=e^{\left[A(t)\right]}\left\{Y(0)\right\}+\int_{0}^{t}e^{\left[A(t-\tau )\right]}\left[B\right]\left\{F(\tau )\right\}d\tau$$

**Figure 3.**
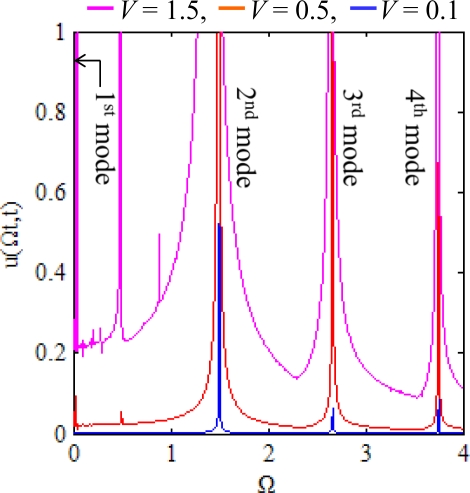
The frequency response of the ring at the location of traveling electrostatic force.

We adopt the first four modes expansion (*n* = 1 ∼ 4) and the commercial software MATLAB for numerical integration. Figure 3 shows the response spectrum of the ring at the location of traveling electrostatic force. Apparently there are four critical speeds, Ω_cr,*k*_ = ω*_k_*/*k*, corresponding to the first four natural modes of the ring. However some peaks other than the natural modes of the ring appear. Therefore, apart from resonance, the ring may be unstable at some unstable traveling speeds.

## 5. Stability Analysis

According to the results of dynamic response, the ring driven by a traveling electrostatic force may be unstable or resonant at the critical speeds of the electrostatic force. Therefore, we have to consider the dynamic instability. Let us consider the homogeneous part of Equation (37), namely:

(42) $${\left\{\dot{Y}\right\}}_{4n\times 1}={\left[A\right]}_{4n\times 4n}{\left\{Y\right\}}_{4n\times 1}$$

where [**A**] is with the period of *T*. Set 4*n* linear independent initial conditions:

(43) $${\left\{Y(0)\right\}}_{1}=\left\{\begin{array}{c} 1\\ 0\\ 0\\ \vdots \\ 0 \end{array}\right\},\;{\left\{Y(0)\right\}}_{2}=\left\{\begin{array}{c} 0\\ 1\\ 0\\ \vdots \\ 0 \end{array}\right\},\;\cdots ,\;{\left\{Y(0)\right\}}_{4n}\left\{\begin{array}{c} 0\\ 0\\ \vdots \\ 0\\ 1 \end{array}\right\}$$

By the Runge-Kutta numerical integration method, we obtain 4*n* linear independent homogeneous solutions of Equation (42) in one period *T*, namely {**Y**(*T*)}_1_, {**Y**(*T*)}_2_, …, {**Y**(*T*)}_4*n*_. The linear independent homogeneous solutions construct the monodromy matrix [**C**], also known as state transition matrix, *i.e.*,

(44) $${\left[C\right]}_{4n\times 4n}=\left[\begin{array}{cccc} {\left\{Y(T)\right\}}_{1} & {\left\{Y(T)\right\}}_{2} & \cdots & {\left\{Y(T)\right\}}_{4n} \end{array}\right]$$

**Figure 4.**
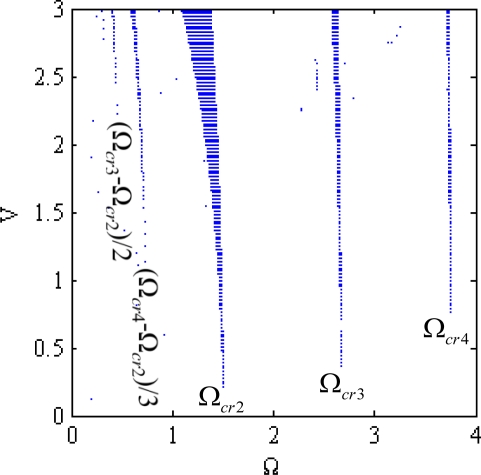
The instability of a micro ring driven by a traveling electrostatic force with the traveling speed of Ω and the driving voltage of *V*.

The stability can be determined by the nature of the eigenvalues (λ) of the monodromy matrix [**C**] [9,10]. The system is stable if all the eigenvalues have magnitudes less than unity, *i.e.*, |*λ*| < 1, unstable if at least one eigenvalue greater than unity, *i.e.*, |*λ*| > 1, and marginally stable if at least one eigenvalue with unit magnitude and multiplicity less than unity. Figure 4 shows the numerical results of stability analysis in the first three flexible modes region. The instable regions appear not only near Ω_cr,*k*_ = ω*_k_*/*k* but also near some fractions of critical speeds differences, say (Ω_*cr*,3_ − Ω_*cr*,2_)/2, (Ω_*cr*,4_ − Ω_*cr*,2_)/3, …*etc*. Furthermore the instable regions expand with increasing driving voltage.

## 6. Conclusions

This paper presents a simplified analytical model for a micro-ring driven by a concentrated electrostatic force which is traveling around its circumference in a radial direction based on the small deflection assumption. We study the dynamics and stabilities of the micro-ring based on the present analytical model and find some interesting phenomena. The micro-ring will resonate when the traveling speeds approach some critical values which are exactly equal to the ratio of the natural frequency to the wave number of each natural mode of the micro-ring. Besides, it will be unstable when the combination of the traveling speed and driving voltage approaches some values. The unstable regions appear not only near the critical speeds, but also near some fractions of critical speed differences. Furthermore, the unstable regions expand with increasing driving voltage.
